# Reviews and Book Notices

**Published:** 1881-06

**Authors:** 


### Reviews and Book Notices.

Article IX.—The Microscope and Microscopical Technology. A Text-Book for Physicians and Students. By Heinrich Frey, Prof. of Medicine in the University of Zurich. Translated and Edited by Geo. Cutler, m.d. Illustrated with 388 wood engravings, 8vo, pp. 660. Cloth, $6.00. Wm. Wood & Co., New York.

To the student of Histology Prof. Frey is no stranger, and this work hardly requires an introduction. Ably written, it is well translated, almost too literally in places, but with a faithful regard for the author's meaning.

The cuts are numerous and truthful. We have chapters upon theory of the microscope; apparatus for measuring and drawing; tests; directions for using, preparing and mounting objects; directions for examination of blood, lymph, chyle, mucus and pus, and all the tissues normally and pathologically considered.

The chapter upon the urine and urinary apparatus is concise but clear. The Microscopical Examination is too briefly treated however, for a work intended for practitioners. A chapter could well be spared from elsewhere and devoted to this subject.

Following the index, which is exceedingly well arranged, are found the price-lists of the various instrument and objective makers, English, Continental and American.

Articee X.—How to See with the Microscope. By J. Edward Smith, m. d. Illustrated. Pp. 410. Duncan Bros., Chicago. 1881.

In this volume we have, given in an informal and chatty way, a good deal of information about stands, objectives, and general manipulation. There are a number of catalogue illustrations, not all bound in correctly however, for pages 40 and 69 should be transposed. These assist the reader about to purchase, and give a fair comparison of most of our American stands with each other, beginning with those of Zentmayer—who rightly occupies a leading position as regards years of service and merit of instruments—down to the stand devised and arranged by the author, "The Acme." A handy one enough it would seem. To him not supplied with a microscope we would say, read the volume before purchasing, as it may save expense and future regret. J. H. T.

ARTICLE XI.–A MANUAL OF JURISPRUDENCE. By Albert Swayne Taylor, M.D., F.R.S., Fellow Royal College of Physicians, Honorary Member of the Medico-Legal Society of New York, of the Société de Médecine Légale of Paris, etc., etc. Eighth American edition, from the tenth London edition, containing the author's latest notes made expressly for this edition; edited, with additional notes and references, by John J. Reese, M.D., Prof. of Medical Jurisprudence and Toxicology in the University of Pennsylvania, etc. With illustrations on wood. 8vo, pp. 934, half Russia, $6.50. Philadelphia: Henry C. Lea's Son & Co.; 1880.

The reputation of this book being so well established, all the reviewer can do is to recommend it to every student and practitioner as the best treatise on Medical Jurisprudence. It is practical and scientific, and a most valuable contribution to classical medicine. This new edition has been carefully revised and brought up to the present requirements of science. H. D. V.

ARTICLE XII.—THE BACTERIA. By Doctor Antoine Maguin, Licentiate of Natural Science, Chief of the Practical Labors in Natural Histology and the Faculty of Medicine of Lyons, etc. Translated by George M. Sternberg, M.D., Surgeon U. S. A. With illustrations. 8vo, cloth, pp. 228. Boston: Little, Brown & Co., 1880. Jansen, McClurg & Co., Chicago.

A beautiful monograph, illustrated with handsome photo-micrographs and lithographic plates. Bacteria have made such impression on the minds of scientific men; and some allied organisms, as *trichinæ*, bring on such a dreadful practical result, that it is impossible for the physician to ignore them any longer. The present work is a necessary supplement to the best treatise on Botany, while half the book is devoted to the physiology of the subject. It should be found in the library of every practitioner of medicine. H. D. V.

Article XIII.—Medical Diagnosis with Special Reference to Practical Medicine. A Guide to the Knowledge and Discrimination of Diseases. By J. M. Da Costa, m.d., Professor of Practice of Medicine and of Clinical Medicine at the Jefferson Medical College, Philadelphia, etc. Illustrated with engravings on wood. Fifth edition; revised. 8vo, pp. 924. Cloth, $6.00. Philadelphia: J. B. Lippincott & Co. 1881. Jansen, McClurg & Co., Chicago.

Five editions of a work like this within sixteen years speak for themselves. Da Costa's has taken its place by the side of the best manuals used in this country, and is being translated into German. A careful study of this book will enable most young practitioners to make a clear diagnosis of, we may say, all their cases, and avoid the remorseful errors which might otherwise shake their faith in medicine, and blunt their energy forever. Diagnosis is the more important in our days, when law-suits for malpractice are so frequent, and the responsibility of the physician so great.

H. D. V.

Article XIV.—Syphilis and Marriage. Lectures delivered at the St. Louis Hospital, Paris. By Alfred Fournier, Professeur de la Faculté de Médecin de Paris, Médecine de l'Hôpital St. Louis, Membre de l'Académie de Médecine. Translated by P. Albert Morrow, m.d., Physician to the Skin and Venereal Department, New York Dispensary; Member of the New York Dermatological Society; member of the New York Academy of Medicine. 8vo, cloth, pp. 252, $2.00. New York: D. Appleton & Co. 1881. Jansen, McClurg & Co., 117 and 119 State street, Chicago.

The Medical Press in this country and abroad have highly praised this work, which is in fact one that we all need. The views of the author are sound and reliable, and a better study of the subject could not have easily been achieved by any other writer than Dr. Fournier. The translation is faithful, not only conveying the ideas and the facts, but even the style and the peculiarities of the French lecturer. This book, however, is better adapted for physicians than students, on account of the social bearings of the subject. H. D. V.

ARTICLE XV.—THE MANAGEMENT OF CHILDREN. By ANNIE M. HALE, M.D.

This little book contains much valuable information for mothers, for whom especially it was written. So many young women become mothers, and have the care of the physical and mental development of children before they know anything of the hygienic laws that should be carried out if they would give the little ones the best chances of life, that it is necessary they should have some one to appeal to beside the old ladies of the neighborhood. The first part of the book is devoted to the care of children in health, and treats of the following subjects: Food and Sleep; How to Dress Children; How and When they should be Bathed; The Importance of Fresh Air, Sunlight and Exercise; Indigestion. The second part contains a description of the most common diseases to which they are subject and the general symptoms by which the trivial diseases which need only a little domestic treatment, may be distinguished from the more serious ones which require the careful attention of a physician. We can recommend it to mothers, and venture to say if they were more familiar with its contents physicians would have fewer calls.

ARTICLE XVI.—A TEXT-BOOK OF THE PHYSIOLOGICAL CHEMISTRY OF THE ANIMAL BODY, INCLUDING AN ACCOUNT OF THE CHEMICAL CHANGES OCCURRING IN DISEASE. By ARTHUR GAMGEE, M.D., F.R.S., Professor in the Victoria University, Manchester; Brackenbury Professor of Physiology in the Owen's College. With Illustrations. Vol. I., 8vo, cloth, pp. 488; $4.50. London: Macmillan & Co., 1880. Jansen, McClurg & Co., Chicago.

The author's reputation is well established, and this work is certainly the best ever written on the subject; still it will suffice for any one to read a dozen pages of it in order to become convinced that the best authors have only laid down the rudiments of physiology. However, this is probably the key to a better understanding of diseases and a more successful treatment. No earnest student, and no practitioner of medicine, whose philosophy extends beyond *similia similibus curantur*, can afford to be without any notion of physiological chemistry, and Dr. Gamgee has just complied with the requests of practitioners, the world over. The first volume contains the physiological chemistry of the elementary tissues of the organism, the blood, the changes which the blood undergoes in disease (a valuable chapter, but very incomplete), the lymph, chyle and transudations, pus, the connective tissues, the epithelial tissues, the contractile tissues, the nervous tissues, etc. A variety of apparatuses are figured in the text, and most of the experiments referred to are so well described as to facilitate their repetition by any student. We will wait anxiously the second volume, and recommend a careful study of this one, especially to those who are engaged in teaching chemistry, physiology, or pathology. H. D. V.

### Article XVII.—Rumbold's Hygiene of Catarrh.

We believe this the first book on catarrh which is devoted wholly to its hygienic treatment, but we also believe, with the author, that it is one of the most important points to be observed in its treatment. The author has made catarrh a special study for the last twenty years, and that he has been faithful and thorough in his study none can doubt who read his book. The first part of the book treats of Hygienic Measures. The importance of preventing colds. The care of the head—its protection day and night. He does not believe in shampooing, for it makes the scalp dry and the hair brittle, but thinks the hair should be oiled enough to keep it moist. The neck should not be bundled up with furs and comforters. Clothing should be thick in winter. Girls are more subject to catarrh than boys of the same age, because they are not warmly dressed, but the ratio changes when they become adults, for then boys take up the tobacco habit. Too frequent bathing and changing of underwear is a frequent cause of colds. The body, like the scalp, should be moist and not too dry. He describes the

"Catheter Nasal Douche," an instrument devised by himself, which, from the description and illustration, must be more effective in removing the crusts from the superior and posterior parts of the nasal cavities than any instrument yet invented. The jets of spray are thrown from the catheter in such a way that they must come in contact with the hard crusts that collect in those regions. We have not examined the instrument, but have no doubt of its usefulness. The Lucas Ear Injector, with his improvement, is a very convenient and ingenious instrument. The gutta percha ring which is molded to fit the outlet of the auditory canal prevents the escape of the fluid, except through the recurrent tube which conducts it into a vessel. The patient can use it himself and keep up a gentle continuous injection, without any danger to the membrana tympani. But one of the best chapters in the book is the one on the mental and physical effects of tobacco. We have suffered ourselves for several years from the injurious effects of the habit, and we can vouch for many of the facts stated. We believe it almost impossible to cure a patient of catarrh while he is continually using the weed. It is like attempting to cure the mucous patches of the mouth of a person with syphilis, who chews tobacco continually. In the fourth division of the chapter he says "the congestion occasioned by the action of tobacco on the mucous membrane of the superior portion of the respiratory tract resembles in many respects the congestion resulting from the effects of a cold, and, like it, some of its effects are transitory and some permanent." We can recommend this book to the profession, for the disease is so common in this climate and so hard to cure, it behooves us all to be as bright as possible on the subject.

ARTICLE XVIII.—THE BLACK ARTS IN MEDICINE, WITH ANNIVERSARY ADDRESS. By JOHN D. JACKSON, A.M., M.D. Edited by L. S. McMURTRY, A.M., M.D., 12mo, pp. 74, cloth. Cincinnati: Robert Clarke & Co., 1880.

From time immemorial, the subject of the first part of this book has formed an interesting topic with medical men; for, although all physicians advertise, only the fool or the ignorant do it in the manner spoken of in this monograph. A great many ways are open to physicians to build their reputation: 1. Talents and skill. Let a man prove himself a learned scholar, an eminent man of science; let him secure a professorship in a medical college, fullfil his position better than any body else could; let him become a favorite with his colleagues, and soon his reputation will extend to the whole community. Then let him write some classical work on some branch of medicine, and his reputation is built and advertised for life. This is a very commendable way of advertising. 2. Honesty and social position. It is in the nature of our vocation to be sociable, honest, reliable citizens, and he who possesses these merits cannot fail to command a large practice wherever he becomes known. It is a subject of grief for the profession at large to be so little represented in politics, religion, or industry, and a large source of income is cut off from its members on that account. Even mental income, if we so style the expansion which the mind receives from a daily contact with all the most important facts of political economy. So much so that we have all met bright young men cheerfully entering the career of medicine, full of energy and vigor, who were soon complete wrecks, so quaint and modeled by prejudices was the profession in the places where they began practice. 3. Professional men must be universal in their knowledge. Lack of this is a great drawback to many practitioners who never studied anything but what is taught in common schools, took two courses of lectures in a medical college, and set to practice. Some of these are remarkable for their ignorance, especially of anything beyond their materia medica, and the routine of practice, and the medical profession, on their account, is looked upon as something insignificant and of doubtful merit.

Let us all advance our standard in these various directions, and the people at large will soon learn to whom to apply for reliable medical attendance, and their money will not be wasted on quacks as much as it is now. Many deficiencies stand in opposition to these views. Indolence on the part of some able men who could assume and fill higher positions; a lack of business in many famous physicians, who engage in ruinous competition with younger members by charging the lowest fees; and last, not least, a sort of ostracism to which most physicians willfully submit. H. D. V.

